# A real-world evaluation of a tertiary care childhood obesity intervention to reduce metabolic risk in a hard-to-reach urban population

**DOI:** 10.1186/s12887-019-1763-5

**Published:** 2019-10-24

**Authors:** Nagla S. Bayoumi, Elizabeth Helzner, Aimee Afable, Michael A. Joseph, Sarita Dhuper

**Affiliations:** 1 Department of Epidemiology & Biostatistics, SUNY Downstate Medical Center, School of Public Health, 450 Clarkson Avenue, Brooklyn, NY 11203 USA; 2 Department of Community Health Sciences, SUNY Downstate Medical Center, School of Public Health, Brooklyn, NY USA; 3 Department of Pediatrics, SUNY Downstate Medical Center, Brooklyn, NY USA

**Keywords:** Pediatric obesity, Severe obesity, BMI percentile, Metabolic syndrome

## Abstract

**Background:**

Research on outcomes associated with lifestyle interventions serving pediatric populations in urban settings, where a majority have severe obesity, is scarce. This study assessed whether participation in a lifestyle intervention improved body mass index (BMI) percentile, BMI z-score, blood pressure, and lipid levels for children and adolescents.

**Methods:**

The Live Light Live Right program is a lifestyle intervention that uses medical assessment, nutritional education, access to physical fitness classes, and behavioral modification to improve health outcomes. Data was analyzed for 144 subjects aged 2–19 who participated for a minimum of 12 consecutive months between 2002 and 2016. McNemar tests were used to determine differences in the proportion of participants who moved from abnormal values at baseline to normal at follow-up for a given clinical measure. Paired sample t-tests assessed differences in blood pressure and lipid levels. Multiple linear regression assessed the change in blood pressure or lipid levels associated with improvement in BMI%_95_ and BMI z-score.

**Results:**

The majority were female (62.5%), mean age was 9.6, and 71% were Black. At baseline, 70.1% had severe obesity, systolic hypertension was present in 44, and 13.9% had diastolic hypertension. One-third had abnormally low high-density lipoprotein (HDL) at baseline, 35% had elevated low-density lipoprotein (LDL), and 47% had abnormal total cholesterol (TC). The average difference in percentage points of BMI%_95_ at follow-up compared was − 3.0 (95% CI: − 5.0, − 1.1; *p* < 0.003). The mean difference in BMI z-score units at follow-up was − 0.15 (95% CI: − 0.2, − 0.1; *p* < 0.0001). Participants with systolic or diastolic hypertension had an average improvement in blood pressure of − 15.3 mmHg (*p* < 0.0001) and − 9.6 mmHg (*p* < 0.0001), respectively. There was a mean improvement of 4.4 mg/dL for participants with abnormal HDL (*p* < 0.001) and − 7.8 mg/dL for those with abnormal LDL at baseline (*p* = 0.036). For those with abnormal baseline TC, a one-unit improvement in BMI%_95_ was associated with a 0.61 mg/dL improvement in TC while holding constant age, contact hours, and months since enrollment (*p* = 0.043).

**Conclusions:**

Participation in the program resulted in significant improvements in BMI percentile, BMI z-score, blood pressure, and lipid levels.

## Background

Pediatric obesity is a grave public health concern and its prevalence in the United States increased from 14.6% in 1999–2000 to 17.4% in 2013–2014 [1]. The prevalence in Brooklyn, New York is greater - approximately 22% of children and adolescents in Brooklyn have obesity [2]. Compared to other regions within Brooklyn, neighborhoods in Central and Eastern Brooklyn have a higher prevalence [2, 3].

Obesity in childhood is associated with elevated blood pressure and abnormal lipid and glucose levels [4]. Childhood obesity tracks into adulthood and 85% of children with obesity become adults with obesity at risk for developing hypertension, type 2 diabetes (T2DM), dyslipidemia, and cardiovascular disease (CVD) [5]. This cluster of diseases and disorders, commonly associated with adulthood, is identifiable in childhood and is known as the metabolic syndrome [5]. Most studies define pediatric metabolic syndrome as the presence of three or more of the following five factors: an increased waist circumference (WC), systolic or diastolic hypertension, a high triglyceride (TG) level, a low high-density lipoprotein (HDL) level, and an elevated fasting glucose concentration [5, 6].

Obesity is a crucial factor for the development of the metabolic syndrome and early identification can help target treatment efforts in high-risk individuals [6] [7]. .Weight loss and its maintenance should have the highest priority in treatment efforts since weight loss has been found to improve blood pressure, serum lipid levels, and fasting blood glucose values [8]. Even a small reduction in body mass index (BMI) percentile or BMI z-score can have beneficial effects on metabolic risk [7]. The treatment of choice for pediatric obesity is a lifestyle intervention focused on weight reduction and based on nutrition education, exercise, and behavioral modification [7, 9–11]. The efficacy of this type of intervention has been proven by several randomized-controlled trials and synthesized in a meta-analyses [11]. The majority of those studies included children with obesity and a gap in the literature exists on the effects of lifestyle interventions on children and adolescents with severe obesity, particularly those residing in urban settings. Our study aimed to fill this gap – the majority of subjects in our study had severe obesity.

Live Light Live Right (LLLR) is a lifestyle modification intervention that combines a set of multi-disciplinary services to help modify behaviors of children with overweight and obesity to lead healthier lives [12]. The program was founded in 2001 and predominantly serves communities in Central and Eastern Brooklyn [12]. Through medical assessment, nutritional education, access to physical fitness classes, and behavioral modification, LLLR aims to improve health outcomes for youth with obesity. The purpose of this study was to determine the impact of the intervention on BMI and metabolic risk factors for children and adolescents, the majority of whom have severe obesity. Specifically, this study aimed to determine whether BMI percentile, BMI z-score, blood pressure, and serum lipid levels improved for enrollees who participated in the LLLR program for a minimum of 12 consecutive months.

## Methods

### Intervention methods

Live Light Live Right is a comprehensive, lifestyle-intervention program. Children between the ages of 2 and 19 with a BMI ≥ 85th percentile for age and sex can be referred to the program. Families are usually referred through a child’s pediatrician, though some learn about the program from social media. Families also learn about the program from screenings that LLLR conducts at public schools, housing complexes, and non-profit organizations in the community.

Program participants were enrolled between January 2002 and August 2016. At the initial visit, a child and his/her parents met with clinic staff and medical histories were obtained. Children underwent a complete medical exam to assess baseline BMI, body composition, waist circumference, and blood pressure measures. Blood samples were taken and lipid levels, liver function, glucose, and insulin levels were determined. A brief psychological screening was administered and referrals for mental health services were arranged when appropriate. Families also met with a nutritionist, who documented dietary habits. A coordinator assessed physical activity habits and time spent in sedentary activity as well as motivation to commit to regular medical visits, counseling sessions, and exercise programs.

The first follow-up visit took place approximately 1 month after the initial appointment. Laboratory test results were reviewed and, based on results of the initial behavioral screenings, a personalized treatment plan specifically tailored to the participant’s needs was developed consisting of four components: 1) regular medical evaluation, 2) nutritional education and counseling, 3) physical activity, and 4) behavioral modification. Each child was assigned a care coordinator who discussed the plan with the child and his/her caretakers, followed up with appointments, monitored participation and attendance to follow-up visits, and facilitated referrals to other community programs. At each subsequent follow-up visit, the plan was modified according to the needs of, and in collaboration with, the participant and his/her family.

During follow-up visits, food recalls were conducted and children and their family members were provided with nutritional education and counseling sessions with certified nutritionists. Among topics discussed within the nutritional education curriculum were: How to Pack Your Pantry, Supermarket Sense, Snacking Savvy, and the Power of Portions. Dietary behavior changes encouraged during counseling sessions included label reading, reducing the intake of fast food and sugary beverages, selecting 3 to 5 servings of fresh fruits and 2 or more servings of vegetables daily, and choosing lean sources of protein. Events such as cooking workshops and age-appropriate games about healthy food choices were scheduled and held at community sites. Program participants and their families were invited to attend and learned where to access fresh and affordable produce and how to prepare nutritious meals.

Treatment plans included, at the minimum, the recommended 60 min of moderate to rigorous daily physical activity. Weekly physical activity recalls were taken at follow-up visits and adherence to plan recommendations were assessed. Participation in structured physical activity was strongly encouraged. The LLLR program partnered with community recreation sites to provide participants with opportunities for free, structured, after-school physical fitness sessions that are supervised by certified trainers. Recognizing that children need a safe and supportive environment to exercise and take part in fitness classes without being ostracized or teased, the sessions were exclusive to LLLR participants. The after-school program provided diversity in sporting choices, including basketball, boxing, aerobics, and dance. In the summer months, participants could attend a six-week day camp that included physical fitness activities as well as education about food insecurity and field trips to community gardens to learn about the benefits of eating fruits and vegetables.

When follow-up visits were missed, barriers were addressed and goals were modified as needed. Motivational interviewing was used to help program participants and their caregivers determine priorities, consider whether current behaviors support priorities, and assess barriers and resources that could influence behavior change. Additionally, the behavioral modification techniques of stimulus control and role-playing were used to encourage healthier dietary and activity choices.

The State University of New York Downstate Medical Center Institutional Review Board approved this study. Assent and written informed consent were obtained from participants and their parents/guardians, respectively.

### Study methods

#### Characteristics of the study sample

**Fig. 1:**
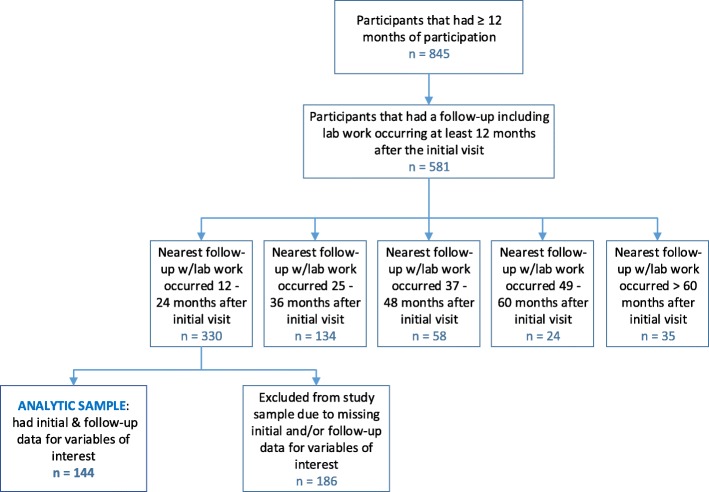
Selection of Analytic Sample (Please see attached file)

This study was a single arm, retrospective, pre-post analysis. Data was analyzed for subjects aged 2 through 19 who participated in the LLLR program for a minimum of 12 consecutive months between the years of 2002 and 2016. To be included in the sample, a follow-up visit with a complete medical reassessment after 12 consecutive months of enrollment had to have occurred anytime between 12 and 24 months after the initial visit. During the study period, 845 children participated in LLLR for a minimum of 12 months. Of these, 144 met the above inclusion criteria and had no missing values for any variables of interest (Fig. 1). Though the analytic sample was slightly younger in age as compared to the overall LLLR cohort, there were no significant differences in regards to sex, BMI%_95_, BMI z-score, and obesity prevalence (Table 1).

**Table 1:** Baseline descriptive statistics of participants with ≥12 months of participation (*n* = 845), participants with ≥12 months of participation that did not have a follow-up that included lab work at least 12 months after the initial visit (*n* = 264), participants with ≥12 months of participation and a follow-up reassessment including lab work at least 12 months after the initial visit (*n* = 581), and the analytic sample which included participants whose nearest follow-up with lab work occurred 12–24 months after the initial visit and had no missing data (*n* = 144)

	Participants with ≥ 12 months of participation *n* = 845	Did not have a follow-up with lab work at least 12 months after initial visit *n* = 264	Had a follow-up with labs at least 12 months after initial visit *n* = 581	Analytic Sample: nearest follow-up with labs occurred 12–24 months after the initial visit and had no missing data *n* = 144
Variable	N (%)	N (%)	N (%)	N (%)
Age in years mean ± SD	10.5 ± 3.5*	11.3 ± 3.5*	10.1 ± 3.4*	9.6 ± 3.0*
Sex				
Male	340 (40%)	113 (43%)	228 (39%)	54 (37.5%)
Female	504 (60%)	150 (57%)	353 (61%)	90 (62.5%)
BMI%_95_ mean ± SD	136.8 ± 25.9	135.7 ± 25.2	137.1 ± 25.9	133.6 ± 22.7
BMI z-score mean ± SD	2.5 ± 0.5	2.4 ± 0.4	2.6 ± 0.5	2.5 ± 0.6
Obesity Prevalence^a^				
Healthy weight	2 (0.2%)	0 (0.0%)	2 (0.3%)	1 (0.7%)
Overweight	20 (2.4%)	8 (3.0%)	12 (2.1%)	2 (1.4%)
Obese	202 (23.9%)	68 (25.8%)	136 (23.4%)	40 (27.8%)
Severely Obese	604 (71.5%)	180 (68.2%)	424 (73.0%)	101 (70.1%)
Missing	17 (2.0%)	8 (3.0%)	7 (1.2%)	0 (0.0%)

Overweight: BMI between the 85th & 94th percentile for age & sex

Obese: BMI ≥ 95th percentile & < 120% of the 95th percentile for age & sex

Severely Obese: BMI ≥ 120% of the 95th percentile for age & sex

*Significant differences between groups; *p* < 0.05

^a^ Healthy weight: BMI between the 5th & 84th percentile for age & sex

### Measurement of outcomes

Anthropometric measures and health indicators were assessed at baseline and at follow-up. All follow-up measures refer to those taken at the first follow-up visit that included a complete medical reassessment after at least 12 consecutive months of program enrollment. Height and weight were measured with the participant in light clothing without shoes. Height was measured to the nearest tenth of an inch using a Detecto stadiometer. Weight was measured to the nearest tenth of a pound using an InBody 270 Body Composition Analyzer machine. The InBody 270 calculated BMI as weight in kilograms divided by the square of height in meters. BMI was then used to determine age and sex-specific BMI z-scores and percentiles using electronic health record calculators. Waist circumference was measured to the nearest tenth of a centimeter just above the iliac crest using a tape measure. Automated blood pressure measurement devices (Welch Allyn 4200B-E1 Vital Signs Monitor and Mindray Passport V Monitor) were used to measure resting systolic blood pressure (SBP) and diastolic blood pressure (DBP). Blood pressure was measured while participants were in the sitting position with the right arm at rest. Participants sat quietly for a few minutes before the first measurement was taken. Three measurements were taken and the average was used. Readings were recorded to the nearest integer in mmHg units. Fasting blood samples were collected by venipuncture and included measures for glucose, insulin, total cholesterol (TC), HDL cholesterol, low-density lipoprotein (LDL) cholesterol, and triglycerides (TG).

To assess the burden of disease at baseline amongst sample participants, the age- and sex-specific prevalence of overweight, obesity, and severe obesity was determined. Overweight was defined as having a BMI between the 85th and 94th percentile. Obese was defined as having a BMI ≥ 95th percentile but < 120% of the 95th percentile. Severe obesity was defined as a BMI ≥ 120% of the 95th percentile. Abnormalities for WC, blood pressure, and lipid levels were determined based on reference values from the National Cholesterol Education Program’s Pediatric Panel report [13, 14]. Waist circumference ≥ 90th percentile for age and sex was defined as abnormal. The presence of hypertension was defined as SBP or DBP ≥ 90th percentile for age, sex, and height. Abnormal lipid levels were defined as follows: TC ≥ 160 mg/dL; HDL ≤ 40 mg/dL; LDL ≥ 110 mg/dL; and TG ≥ 110 mg/dL for those 12 yrs. and older or ≥ 90th percentile for age and sex. An abnormally high glucose level was defined as ≥110 mg/dL. Elevated metabolic risk was defined as the presence of three or more of the following factors: an abnormally large WC, systolic or diastolic hypertension, an abnormally low HDL level, an abnormally high TG level, and an elevated fasting glucose level.

### Measurement of covariates

Demographic factors including age, gender, race and ethnicity were measured at baseline. Contact hours with the LLLR program were used as a proxy for treatment intensity. Contact hours included time spent at the clinic for initial visits, follow-up visits, nutrition education, nutritional counseling, physical activity education, and behavioral counseling. The number of months of program participation was calculated as the number of months enrolled since the initial visit.

### Statistical methods

Data were analyzed using IBM SPSS software version 24 [15]. McNemar tests were used to determine differences in the proportion of participants who moved from abnormal values at baseline to normal at follow-up for a given clinical measure. Paired sample t-tests were used to determine whether mean differences in anthropometric measures, blood pressure, lipid levels, and glucose measures were significantly different at follow-up compared to baseline. Binary logistic regression models were used to determine the odds associated with a one-unit improvement in BMI%_95_ and BMI z-score on the normalization of blood pressure and lipid levels while controlling for age, sex, contact hours, and months since initial visit. Multiple linear regression models were used to assess the change in blood pressure or lipid levels associated with a one-unit improvement in BMI%_95_ and BMI z-score while controlling for age, contact hours, and months since initial visit. The significance level was set at alpha = 0.05.

## Results

Descriptive statistics of the study sample at baseline are shown in Table 2. The majority of participants were female (62.5%) and the mean age was 9.6 years. About 71% of the sample identified as Black and 16.7% were Hispanic. The majority of participants had severe obesity (70.1%). Table 3 shows the number and percentage of participants with abnormal WC, blood pressure, lipid levels, and fasting glucose measures at baseline. The majority of participants had an abnormal waist circumference (92.4%). At baseline, systolic hypertension was present in 44 and 13.9% had diastolic hypertension. One-third of the sample had abnormally low HDL levels at baseline and 25.7% had elevated TG levels. Approximately 5% had an elevated fasting glucose level at baseline. About 31% of sample subjects (*n* = 44) were identified as having three or more components of the metabolic syndrome at baseline.

**Table 2:** Descriptive statistics of sample (*N* = 144) at baseline

Variable	N (%)
Age in years, mean ± SD	9.6 ± 3.0
Sex	
Male	54 (37.5%)
Female	90 (62.5%)
Race	
Black	102 (70.8%)
Hispanic	24 (16.7%)
White	1 (0.7%)
Asian	1 (0.7%)
Other/Unknown	16 (11.1%)
Obesity Prevalence^a^	
Healthy Weight	1 (0.7%)
Overweight	2 (1.4%)
Obese	40 (27.8%)
Severely Obese	101 (70.1%)
Contact hours, mean ± SD	7.6 ± 4.1
Months enrolled since initial visit, mean ± SD	17.2 ± 3.7

Overweight: BMI between the 85th & 94th percentile for age & sex

Obese: BMI ≥ 95th percentile & < 120% of the 95th percentile for age & sex

Severely Obese: BMI ≥ 120% of the 95th percentile for age & sex

^a^Healthy weight: BMI between the 5th & 84th percentile for age & sex

**Table 3:** The number and percentage of participants with abnormal measures at baseline and follow-up and the number and percentage with normalized measures at follow-up of those with abnormal baseline measures

Measure	Abnormal at Baseline N (%)	Normalized measure at follow-up of those with abnormal measure at baseline N (%)	Abnormal at Follow-up	*p* value*
Waist Circumference^a^	133 (92.4%)	10 (7.5%)	127 (88.2%)	*p* = 0.180
Blood Pressure^b^				
Systolic blood pressure	64 (44.4%)	33 (51.6%)	55 (38.2%)	*p* = 0.289
Diastolic blood pressure	20 (13.9%)	18 (90.0%)	15 (10.4%)	*p* = 0.473
Lipid Levels^c^				
Total cholesterol	67 (46.5%)	16 (23.9%)	65 (45.1%)	*p* = 0.856
High-density lipoprotein	48 (33.3%)	22 (45.8%)	38 (26.4%)	*p* = 0.121
Low-density lipoprotein	50 (34.7%)	13 (26.0%)	54 (37.5%)	*p* = 0.585
Triglycerides	37 (25.7%)	18 (48.6%)	39 (27.1%)	*p* = 0.871
Fasting Glucose^d^	7 (4.9%)	6 ((85.7%)	3 (2.1%)	*p* = 0.289
Presence of 3 or more components of the metabolic syndrome^e^	44 (30.6%)	22 (50.0%)	39 (27.1%)	*p* = 0.522

*p-values refer to McNemar tests

^a^Waist circumference ≥ 90th percentile for age and sex was defined as abnormal [14]

^b^Hypertension was defined as SBP or DBP ≥ 90th percentile for age, sex, and height [14]

^c^Abnormal lipid levels were defined as: TC ≥ 160 mg/dL; HDL ≤ 40 mg/dL; LDL ≥ 110 mg/dL; and TG ≥ 110 mg/dL for those 12 yrs. and older or ≥ 90th percentile for age and sex [14]

^d^Abnormally high fasting glucose level was defined as ≥110 mg/dL [14]

^e^Defined as presence of 3 or more of the following: increased waist circumference, systolic or diastolic hypertension, a high TG level, a low HDL level, or elevated fasting glucose concentration

At follow-up, approximately 62% of participants experienced a reduction in or maintenance of BMI%_95_ and 68% had a reduction in or maintenance of BMI z-score. Mean differences between follow-up and baseline measures of BMI%_95_ and BMI z-score for the entire sample are displayed in Table 4. There were significant reductions in both BMI%_95_ and BMI z-score. The average difference in percentage points of BMI%_95_ at follow-up compared to baseline was − 3.0 (95% CI: − 5.0, − 1.1; *p* < 0.003). The mean difference in BMI z-score units at follow-up compared to baseline was − 0.15 (95% CI: − 0.2, − 0.1; *p* < 0.0001).

**Table 4:** Change in mean BMI%_95_ and BMI z-score for the entire study sample

Measure	Baseline Mean ± SD	Follow-up Mean ± SD	Difference (Δ) Mean (95% CI)	*p* value*
BMI%_95_	133.6 ± 22.7	130.6 ± 24.2	−3.0 (− 5.0, − 1.1)	*p* < 0.003
BMI z-score	2.5 ± 0.6	2.4 ± 0.5	− 0.15 (− 0.2, − 0.1)	*p* < 0.0001

* p-values refer to paired-sample t-tests

Table 5 shows changes in mean blood pressure, lipid levels, and glucose measures for participants who had abnormal levels at baseline. Participants with systolic or diastolic hypertension at baseline had an average improvement in blood pressure of − 15.3 mmHg (*p* < 0.0001) and − 9.6 mmHg (*p* < 0.0001), respectively. Significant improvements in HDL and LDL were also observed for participants who had abnormal levels at baseline. At follow-up, there was a mean improvement of 4.4 mg/dL for participants with abnormal HDL levels at baseline (*p* < 0.001) and − 7.8 mg/dL for those with abnormal LDL levels at baseline (*p* = 0.036).

**Table 5:** Change in mean blood pressure, lipid levels, and fasting glucose measures for participants who had abnormal levels at baseline

Measure	Baseline Mean ± SD	Follow-up Mean ± SD	Difference (Δ) Mean (95% CI)	*p* value*
SBP (mm Hg)	122.6 ± 7.6	117.3 ± 11.3	−15.3(− 7.7, − 2.9)	*p* < 0.0001
DBP (mm Hg)	77.1 ± 7.7	67.6 ± 10.7	−9.6 (− 13.5, − 5.6)	*p* < 0.0001
TC (mg/dL)	196.0 ± 25.4	192.3 ± 32.0	−3.7 (− 9.8, 2.3)	*p* = 0.221
HDL (mg/dL)	36.2 ± 4.6	40.6 ± 8.8	4.4 (2.0, 6.8)	*p* < 0.001
LDL (mg/dL)	134.5 ± 19.4	126.7 ± 29.2	− 7.8 (− 15.1, − 0.55)	*p* = 0.036
TG (mg/dL)	140.8 ± 58.3	122.0 ± 49.2	−18.9 (− 41.8, 4.0)	*p* = 0.103
Glucose (mg/dL)	120.7 ± 27.4	91.7 ± 7.7	−29.0 (− 3.9, 0.99)	*p* = 0.054

* *p*-values refer to paired-sample t-tests

Results of the McNemar tests (Table 3) did not yield significant differences in the proportion of participants who moved from abnormal values at baseline to normal at follow-up for a given clinical measure. In binary logistic regression analyses using normalization of blood pressure and lipid levels as outcomes, change in BMI%_95_ and BMI z-score were not associated with greater odds of normalization except in the case of TC (Table 6). For those with abnormal TC levels at baseline, a one unit increase in BMI%_95_ was associated with a 7% reduced odds of normalized TC levels at follow-up while adjusting for age, sex, contact hours, and months since initial visit (OR = 0.93, 95% CI: 0.87, 0.99, *p* = 0.03). Except for TC, multiple linear regression analyses did not yield significant associations between reductions in BMI%_95_ and BMI z-score and improvements in blood pressure or lipid levels (Table 7). For those with abnormal baseline TC, a one-unit improvement in BMI%_95_ was associated with a 0.61 mg/dL improvement in TC while holding constant age, contact hours, and months since enrollment (*p* = 0.043).

**Table 6:** Logistic regression model results with normalized total cholesterol at follow-up as the dependent variable

Model variables^a^	β	*p* value	Exp(β)	95% CI
Dependent variable: Normalized TC				
Change in BMI%_95_	−0.076	*p* = 0.032	0.927	(0.865, 0.994)
Age	0.015	*p* = 0.869	1.016	(0.845, 1.220)
Sex (male)	− 0.058	*p* = 0.927	0.943	(0.270, 3.301)
Contact hours	− 0.029	*p* = 0.749	0.971	(0.811, 1.163)
Months since initial visit	− 0.013	*p* = 0.873	0.987	(0.842, 1.157)

^a^All variables were entered simultaneously into the model

**Table 7:** Multivariable linear regression model results with change in total cholesterol at follow-up as the outcome variable

Model variables^a^	β	*p* value
Outcome variable: Change in TC (mg/dL) at follow-up		
Change in BMI%_95_	0.608	*p* = 0.043
Age	1.124	*p* = 0.213
Contact hours	0.245	*p* = 0.790
Months since initial visit	0.084	*p* = 0.915

^a^All variables were entered simultaneously into the model

## Discussion

Participation in the LLLR program for a minimum of 12 consecutive months resulted in significant improvements in components of the metabolic syndrome. In our study, 50% of participants who had three or more components of the metabolic syndrome at baseline had less than three components at follow-up. In the literature, lifestyle interventions for children and adolescents have been associated with a significant decrease in the prevalence of the metabolic syndrome. A study conducted by Reinehr et al. found that the prevalence of the components of the metabolic syndrome decreased significantly in 288 children with obesity after a 1-year lifestyle intervention in contrast to the 186 children in the control group without a lifestyle intervention [16]. The intervention resulted in a significant decrease of metabolic syndrome prevalence of 19 to 9% [16]. Similarly, Verduci et al. reported that a 1-year behavioral intervention for children with obesity resulted in a significant decrease of 17 to 5% from baseline to the end of the intervention [17]. The difference in proportion in our study between the percentage with three or more components of the metabolic syndrome at follow-up compared to baseline was not significant. This might be partly explained by the fact that, unlike the two studies described above, a majority of participants had severe obesity and a greater percentage (30.6%) had three or more components of the metabolic syndrome at baseline.

The findings of our study are similar to those of a study by Wickham et al. that included 165 youth with obesity of which 30.3% had three or more components of the metabolic syndrome at baseline [18]. After 6 months of lifestyle modification, Wickham et al. did not find a significant difference in the number of subjects with three or more criteria of the metabolic syndrome [18]. Similar to our study, 70.3% of the participants in the study by Wickham et al. were African American and the mean BMI z-score at baseline was 2.44 (± 0.31) [18]. Lifestyle modification programs vary in intensity, which explains variations in results amongst studies. We agree with Wickham et al. who reasoned that more intense exercise programs were required to see significant changes in metabolic syndrome prevalence in certain patient populations [18].

After participation in the LLLR program for a minimum of 12 consecutive months, significant differences in SBP, DBP, HDL, and LDL were observed for participants with abnormal baseline levels. Wickham et al. did not find significant differences in mean pre/post blood pressure measures though TC and LDL decreased significantly from baseline [18]. In a study of 177 youth with obesity aged 5 to 19 who took part in a behavioral weight management program, Kirk et al. found significant improvements in blood pressure and LDL levels at follow-up for subjects who had abnormal levels at program onset, though improvements in TC were also observed [19]. In a retrospective cohort study of 282 2- through 19-year old youth with obesity who participated in a primary care-based childhood obesity treatment program, Dolinsky et al. reported significant improvements at follow-up for patients with elevated SBP, DBP, TC, and TG levels [20].

A meta-analysis of 15 studies found that lifestyle interventions led to significant improvements in LDL levels (− 5.4 mg/dL, 95% CI: − 8.1, − 2.7), TG levels (− 2.7 mg/dL, 95% CI: − 4.32, − 1.26), and blood pressure up to 1 year from baseline though no difference was found for HDL levels [21]. In comparison, our study found significant mean differences in pre/post measures of blood pressure and LDL levels though it is important to note that a significant mean difference in HDL for those with abnormal levels at baseline was also observed. In addition to improvements in TG levels, Verduci et al. found significant increases in HDL levels at the end of the 1-year intervention (1.1 mg/dL, 95% CI: 0.2, 2.0) though the magnitude in the difference of HDL found in our study was greater [17].

Although there were significant mean differences between follow-up and baseline measures of BMI%_95_ and BMI z-score, associations between reductions in BMI%_95_ and BMI z-score and improvements in blood pressure or lipid levels were only significant for TC. This is most likely due to relatively fewer numbers of participants with abnormal baseline measures for blood pressure, HDL, LDL, and TG compared to TC. Some studies reported associations between decreases in BMI z-score and improvements in blood pressure or lipid levels and others did not. The meta-analysis referenced above reported that improvements in lipid levels were not uniformly associated with the extent of weight loss or body fat reduction and that it was unclear whether positive effects were attributable to weight loss per se or to factors of lifestyle interventions independent of weight loss, such as an increase in physical activity or a reduction in saturated fat intake [21]. This is important to note considering studies have reported that lifestyle interventions in children with obesity have resulted in improvements in blood pressure and lipid levels with the maintenance of BMI z-score and in the absence of weight loss or body composition change [21–24].

This study had some limitations. The number of contact hours included time spent in the clinic on medical assessments, reassessments, behavioral modification, education, and counseling. It did not include time spent at offsite program-sponsored physical activity sessions. Future research should assess the impact of participation in program-sponsored fitness classes on the components of the metabolic syndrome. The lack of a randomized control group was another limitation and, although that would have been optimal, it was unlikely that untreated youth with obesity would improve their relative weight status or metabolic risk profile. Although baseline characteristics of the analytic sample were similar to those of the entire sample, the analytic sample was slightly younger. As a result, there was a potential for selection bias. Findings of this study are not generalizable to all LLLR participants since the presence of a complete follow-up reassessment and when that follow-up took place relative to the initial visit could be influenced by factors not controlled for, such as motivation. Therefore, findings are generalizable to participants with similar characteristics to the analytic sample. The use of an intervention duration of 12 months, at minimum, and 24 months, at maximum, precluded the assessment of longer-term results. Future follow-up studies on the sample will determine if improvements in blood pressure and lipid levels are maintained over longer durations.

Despite these limitations, confidence in our findings is strengthened by the variety of outcome indicators used to assess intervention effectiveness. We assessed pre/post differences for blood pressure, lipid levels, and other components of the metabolic syndrome whereas many studies looked at either changes in blood pressure or lipid levels, but not both. Another strength was the definition of severe obesity as BMI ≥ 120% of the 95th percentile. As this definition for severe obesity becomes used more widely in research on extreme obesity in pediatric populations, we expect our findings will be useful for comparative purposes. Data about improvements in cardiovascular disease risk factors related to obesity interventions for children and adolescents residing in Central and Eastern Brooklyn are scarce. This study adds to the research on a population that is gravely underrepresented within the literature and our findings have implications for the benefits of lifestyle interventions for youth with severe obesity.

## Conclusion

Participation in the LLLR program resulted in reductions in BMI%_95_ and BMI z-score and significant improvements in blood pressure and lipid levels for participants with abnormal baseline measures. Since obesity is a chronic disease requiring ongoing care, the evaluation of long-term outcomes of the program are recommended to determine if improvements are sustained. When resources and assets are mobilized strategically, a community-based approach to the treatment of pediatric obesity can directly affect the health and well-being of children and adolescents.

## Data Availability

The data analyzed during the study Live Light Live Right’s programmatic data and are not publicly available.

## Abbreviations

BMI: Body mass index
CVD: Cardiovascular disease
DBP: Diastolic blood pressure
HDL: High-density lipoprotein
LDL: Low-density lipoprotein
LLLR: Live Light Live Right
SBP: Systolic blood pressure
T2DM: Type 2 diabetes mellitus
TC: Total cholesterol
TG: Triglyceride
WC: Waist circumference
